# Protocol for a pilot cluster randomised controlled trial of a multicomponent sustainable return to work IGLOo intervention

**DOI:** 10.1186/s40814-023-01439-3

**Published:** 2024-02-03

**Authors:** Oliver Davis, Jeremy Dawson, Lizzie Degerdon, Jaime Delgadillo, Umesh Kadam, Karina Nielsen, Alice Sinclair, Jo Yarker, Fehmidah Munir

**Affiliations:** 1 Grounded Research Team, Rotherham Doncaster and South Humber NHS Trust, Doncaster, UK; 2 Sheffield University Management School, University of Sheffield, Sheffield, UK; 3 Department of Psychology, University of Sheffield, Sheffield, UK; 4 Department of Health and Community Sciences, University of Exeter, Exeter, UK; 5 Institute of Work Psychology, University of Sheffield, Sheffield, UK; 6 School of Sport, Health and Exercise Science, Loughborough University, Loughborough, UK; 7 Affinity Health at Work, London, UK

**Keywords:** Return-to-work, Long-term sick leave, Mental health, Intervention, Workplace, Employee, Manager, Employer, Organisation

## Abstract

**Background:**

Long-term sickness costs businesses in the United Kingdom (UK) approximately £7 billion per annum. Most long-term sickness absences are attributed to common mental health conditions, which are also highly prevalent in people with acute or musculoskeletal health conditions. This study will pilot the IGLOo (Individual, Group, Leaders, Organisation, overarching context) intervention which aims to support workers in returning to and remaining in work following long-term sickness absence. The potential impact of the intervention is a timely return to work (main trial primary outcome) and prevention of a further episode of long-term sick leave. The intervention will be piloted in a randomised controlled trial (RCT) to examine the feasibility of the intervention (pilot trial primary outcome) and to inform a fully powered definitive trial to evaluate sustainable return to work (RTW) in people with primary or secondary mental ill-health who go on long-term sick leave.

**Methods and design:**

A two-arm feasibility randomised controlled trial (with a 30-month study period including 12-month follow-up) of the IGLOo intervention will be conducted in large organisations (≥ 600 workers) from the Yorkshire and Humberside regions, in the UK. Eight consenting organisations will be recruited and randomised to the intervention or control arms of the study (1:1 ratio), with a minimum recruitment target of 13 workers eligible to participate from each. Organisations assigned to the control group will continue with their usual practice. Feasibility data will include data collected on recruitment, retention and attrition of participants; completion of research outcome measures; and intervention compliance. Measurements of mental health, RTW, work outcomes, quality-of-life, workplace support and communication and other demographic data will be taken at baseline, 3, 6, 9 and 12 months in all participants. Qualitative interviews and survey data with all participants will explore the experiences of participants, acceptability of the intervention components and evaluation measures. Exploratory economic evaluation will be conducted to further inform a definitive trial.

**Discussion:**

The findings from this pilot study will help to inform the development of a definitive cluster RCT designed to examine the efficacy of this intervention on health and work-related outcomes in UK workers on long-term sick leave.

**Trial registration:**

ISRCTN11788559 (prospectively registered, date registered 6 October 2022)

## Background

Long-term sickness absence costs UK businesses approximately £7 billion per annum [1]. Most long-term sickness absences are attributed to common mental health conditions (e.g. stress, depression, anxiety) [2], which are also highly prevalent in people with acute (e.g. cardio-respiratory, stroke) or musculoskeletal health conditions [2, 3]. Keeping people at work following long-term sick leave is a societal challenge because long-term sick leave is strongly linked to disability pension, unemployment and job termination [4]. With an ageing workforce, the risk of ill-health and life-long disability is also rising [5], bringing further societal challenges. Moreover, the coronavirus SARS-2 (COVID-19) pandemic brings unprecedented difficulties to people’s mental health [6], with global prevalence estimates of mental health issues among the general population amid the pandemic reported to be 28.0% for depression; 26.9% for anxiety and 36.5% for stress symptoms [7]. Furthermore, the prevalence of long-term fatigue, cognitive problems and anxiety and depressive symptoms is evident months after infection [8].

The need for practical measures to enable employer-led sustainable return to work has received considerable attention over the last decade [9]. In 2019, the National Institute for Health and Care Excellence (NICE) [1] highlighted a UK research gap in effective and cost-effective interventions to reduce long-term sickness absence (i.e. those occurring for >14 consecutive days) and supportive return to work particularly for common mental health conditions. Long-term sickness absence not only has a negative impact on employers but also has a significantly detrimental effect on workers [10].

For people returning to work following long-term sick leave, good work can be therapeutic by minimising the harmful effects of long-term sickness absence, loss of work productivity, and the risk of long-term incapacity [11]. However, facilitating a return to work and enabling people to stay at work is challenging, especially where a common mental health condition is the main reason for long-term sick leave or is present for another reason [12–14].

An interplay of factors beyond the health condition is known to impact both return-to-work (RTW) outcomes (defined as the number of sick leave days until the first day of RTW with adjusted working hours or usual working hours) [15] and sustainable RTW outcomes (defined as number of days staying in work over a 6-month period after returning with no exit or long-term sick leave re-occurrence) [16]. Lower education and socio-economic status, older age, lower self-efficacy, poor line manager and/or co-worker support, inadequate work adjustments or lack of freedom to work flexibly (i.e. job crafting) and inadequate workplace return-to-work policies can all hamper sustainable return to work [16–19]. This has a detrimental impact on workers, leading to early retirement, job termination, unemployment [4] and reduced quality of life [9]. It also has a negative impact on employers through sick pay, staff turnover and productivity loss [9] and, more broadly, on society through health-related state benefits.

Evidence suggests that return-to-work outcomes in those with poor mental health are enhanced when symptom/problem-focused interventions (e.g. work-focused Cognitive Behavioural Therapy or solution-focused skills training ) are combined with elements of their workplace (e.g. contact with line manager during absence) [15, 16, 19]. A Cochrane review into mental health and long-term sick leave [19] found moderate evidence that a combination of work-directed and clinical interventions (such as psychological treatment) reduces sickness absence days within the first year of follow-up (SMD −0.25, 95% CI −0.38 to −0.12: 9 studies). Whilst this translates “to 0.5 fewer (95% CI −0.7 to −0.2) sick leave days in the past 2 weeks or 25 fewer days during 1 year (95% CI −37.5 to 11.8)” [19], the authors of the review propose that the integration of clinical and work-directed elements of an intervention is key to improving work outcomes.

In summary, the above studies highlight two important issues: (1) where workers with poor mental health receive a multi-component intervention targeting both work (e.g. line manager support) and the self (e.g. cognitive and affective well-being) they are more likely to return quicker than those who do not receive such an intervention [15, 19, 20], and (2) the type of intervention received by the worker while on sick leave (i.e. multi-component with elements of CBT and with manager support) as well as the *work-related support* received *after they return to work* impacts how long they stay in work without a relapse or long-term sick leave re-occurrence [16, 17]. A review by Philpot et al. [21] suggested that multi-level interventions could be more effective in addressing the aforementioned issues as they build resources at multiple levels and create a synergistic effect for sustainable RTW. For example, by ensuring at the organisational level, there are adequate long-term sick leave and return to work policies that guide the worker and the manager through the return to work process; at the senior level, senior leaders and managers are knowledgeable (and trained) on best practice for managing long-term sick leave and return to work outcomes; at the team level, workers are well-supported when they are back at work by their manager and their colleagues, and the worker has the necessary individual tools to help them return to, and stay at work (e.g. access to tools based on elements of work-focused cognitive behavioural therapy (CBT) and job crafting). However, sustainable return-to-work interventions are in their infancy and more workplace return-to-work research is needed on combined multi-component and multi-level interventions, their effectiveness and the mechanism by which the intervention works [19].

**Fig. 1:**
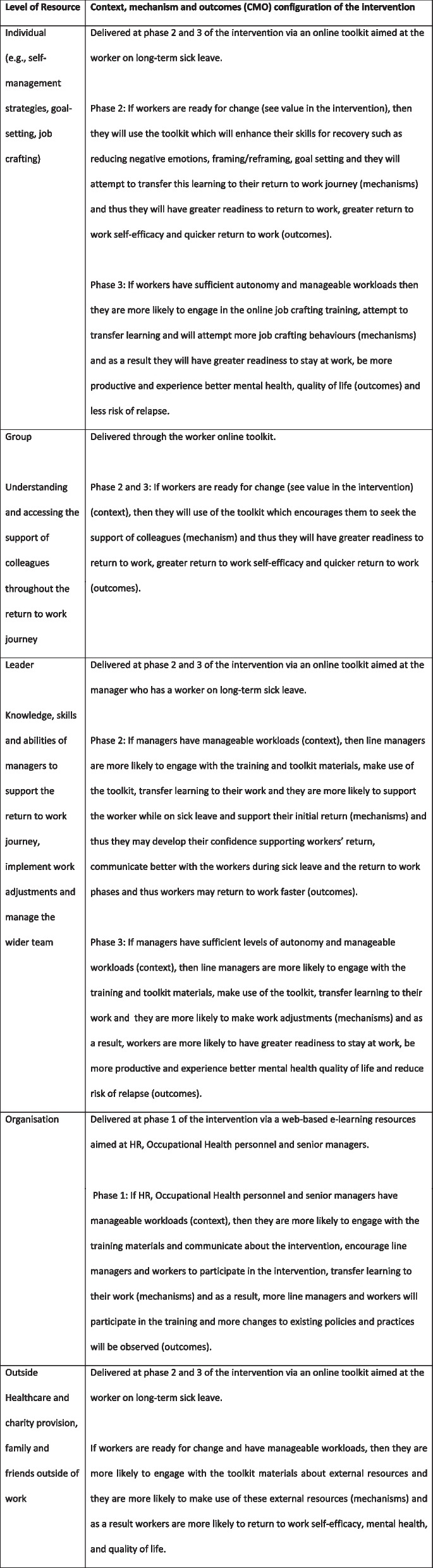
IGLOo model and the hypothetical context, mechanism and outcomes for the IGLOo levels

The IGLOo intervention offers a theoretically led multi-component and multi-level intervention structured around the IGLOo model [17] to promote sustainable return to work in workers. Underpinned by the conservation of resources theory [22], the IGLOo intervention targets five levels of resources to enable a quicker and sustainable return to work outcome (see Fig. 1). These are as follows: Individual resources (e.g. knowledge, skills and CBT-based activities that improve the worker’s self-efficacy to return to work), Group resources (e.g. strategies that enable the worker to access relevant support through colleagues), Leader resources (e.g. conversation guides that help improve manager support), Organisation level (e.g. upskilling senior management and human resources (HR) staff in best practice for return-to-work policies and processes) and the Outside (Overarching) context in the social environment (e.g. raising awareness of local charity provisions for the worker). The intervention components are therefore also underpinned by psycho-social theories Cognitive theory (CT) [23], Communication Accommodation Theory (CAT) [24] and the Socio-Cognitive Theory (SCT) [25].

The IGLOo intervention delivers the resources to workers through an online toolkit (see Fig. 1). Managers are also provided with an online toolkit to guide the return-to-work process and support the workers following their RTW. The guidance and resources in the toolkits for the worker and the manager mirror each other to ensure both receive the same messages and to encourage transparency. The organisational resources are delivered in the intervention by e-learning resources for senior leaders. Nielsen et al. [17] propose workers with common mental health conditions whose experiences of their work group are positive upon RTW are more likely to achieve a sustainable RTW. The aim of this study is to pilot the intervention in a cluster RCT and assess the feasibility of the intervention as the primary outcome, along with the acceptability of a range of mental health and work-related outcome measures to inform a fully powered definitive trial.

## Methods/design

### Study design

**Fig. 2:**
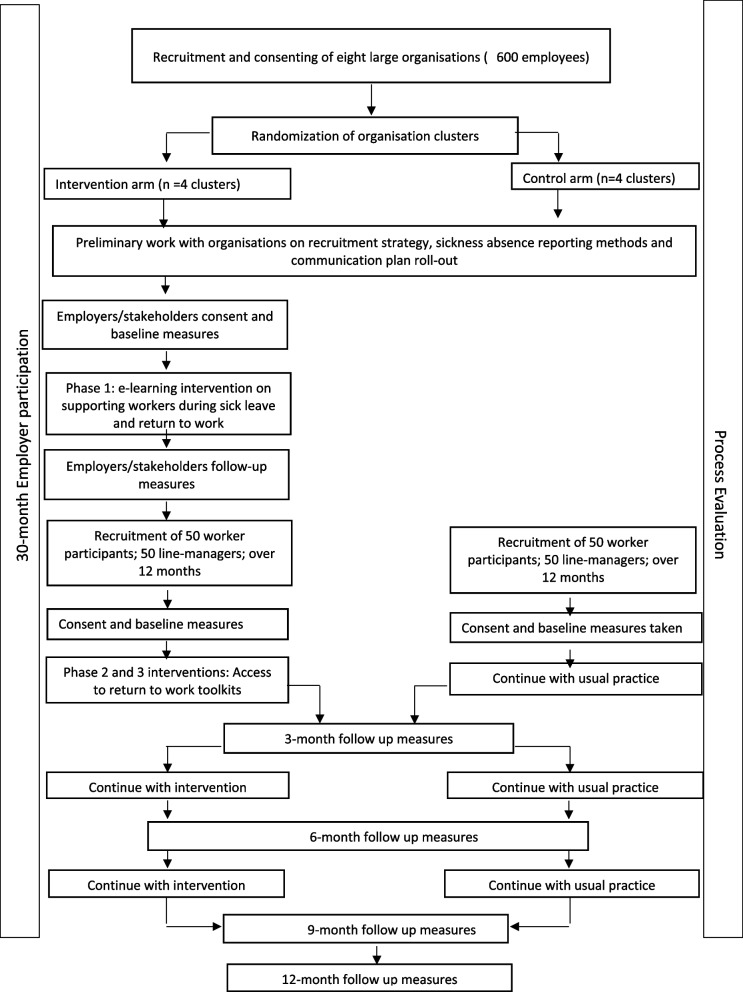
Study flow diagram

The study design follows the UK Medical Research Council guidance for complex interventions [26]. This will be a pilot cluster randomised controlled trial of the IGLOo intervention. The study will last 36 months with organisations participating for 30 months. Within each participating organisation, the recruitment of workers on long-term sick leave will take place over 12 months. Workers will be recruited between >14 and <42 days for their long-term sick leave. Key process outcome measures will be collected monthly through regular meetings with each organisation’s study contact (e.g. key HR staff), while research outcome measures will be collected from each worker and line manager participant at baseline, 3, 6, 9 and 12 months. As a cluster RCT, the unit of randomisation will be the organisation, such that some organisations will receive the pilot intervention and others will not, although the collection of the outcome measures will take place in all participating organisations. Figure 2 shows the study flow diagram, and Table 2 indicates the schedule of enrolment, intervention, and outcome measures. The project has been granted ethical approval by the East Midlands Ethical Advisory Committee. The trial was preregistered in the International Standard Randomised Controlled Trial Number (ISRCTN) registry on 06/10/2022.

### Setting

Employer organisations in the regions of Yorkshire and South Humber, within England. This regional setting has been chosen because of its socioeconomic diversity. For example, 9.4% of its population are from Black, Asian and other minority ethnic backgrounds [27] with 4.7% in Doncaster and 6% in Sheffield describing themselves as ‘non-white’ in the 2011 census [28]. The region also has some of the most socioeconomically deprived areas [28], and with around 75% of 16–64-year-olds in active employment [29], the area has one of the highest rates of sickness absence, at 2.2%, in England [29]. Rates of poor mental health are also disproportionally higher in South Yorkshire and South Humber compared with the national average, with lower self-reported rates of happiness, wellbeing and sense of worthwhile recorded in both Doncaster and Sheffield in 2021 [30]. Some regions in the North of England have been hit especially hard by the COVID-19 pandemic, with higher incidences of COVID-19, higher mortality rates and an evidentially more detrimental impact on the already challenged mental and financial well-being of the population [31].

### Sample size

We will recruit eight cluster organisations [32] with ≥ 600 hundred workers (to minimise the risk of missing recruitment targets) into the feasibility pilot study (randomised in each arm) to calculate the target sample size for a definitive RCT. There is no formal requirement to conduct sample size calculations for pilot trials [33], but a sample of at least 100 participants is desirable [26]. Our target recruitment is 50 workers in the intervention arm and 50 workers in the control arm. We have no recruitment targets set for managers. Approximately 13 workers from each organisation will need to be recruited to reach our sample size. However, we aim to recruit 30 workers and 30 managers from each organisation to participate in the study to ensure that, even with a high possible level of attrition, 50 cases (workers) per arm are achieved [26, 33].

The target population is workers on long-term sick leave due to mental ill health or for a condition known to be associated with mental ill health [2, 12–14], who will be identified by their workplace between > 14 and < 42 days of their long-term sick leave and sent an invitation on behalf of the research team to participate in the study. The line manager of the individual worker on long-term sick leave will also be invited to participate in the study. Participation of the line manager is entirely voluntary, irrespective of whether their worker is taking part.

#### Inclusion criteria

- Large organisations with ≥ 600 workers. This will include NHS trusts, public and private sector employers in the Yorkshire and Humber regions.
- Individuals on long-term sick leave (defined as >14 days) due to occupational burnout and/or a common mental health problem or where is it known as an associated comorbidity [2, 12–14].
- Managers of workers on long-term sick leave.

#### Exclusion criteria

- Organisations that outsource their return-to-work management.
- Organisations that have <2% of workers taking long-term sick in the past 12 months [29].
- Individuals on long-term sick leave due to a severe mental disorder (psychotic disorder; bipolar disorder); substance use disorder; a neurological condition such as dementia; or under investigation for misconduct or formal disciplinary action.
- Workers under 18 years of age.

#### Allocation to intervention

In this pilot RCT, organisations will be randomised using a 1:1 ratio. Randomisation to trial conditions (intervention versus control) will be carried out by assigning pre-prepared envelopes containing the allocation outcomes to the organisation. In each participating organisation, participants (i.e. senior leaders, managers and workers) will partake in the condition their organisation is allocated to and will therefore not be blinded in their group allocation. Prior to the intervention being deployed, an organisation-wide survey will take place to explore the ethos, culture and attitudes towards procedures for managing mental health and return to work in the organisation. All workers at the organisation will be eligible to participate in this.

#### Intervention

Using evidence from the scientific literature and best practice guidelines for the UK (NICE, 2019) [2], the intervention content has been co-created with workers with mental health and RTW experience and managers and employers from both small and medium enterprises (SMEs) and large enterprises (LEs) and encompass the five IGLOo levels across three intervention delivery phases:

*Phase 1* is directed towards the organisational level of the IGLOo model, which includes HR and Occupational Health personnel, senior leaders and others involved in the absence management process in their organisation. Phase 1 participants will be provided with an online e-learning resource hosted on a website consisting of short videos, interactive case studies and learning activities on how leaders can help reduce barriers to workers returning to and staying in work. Modules include the following: (1) Foundations: Mental Health and RTW, which provides education around mental health and its relationship with the workplace. (2) RTW policies and practices, which gives insight into recommended approaches to long-term sickness absence management recommended in the NICE guidelines, and (3) Setting up for success, which involves how to integrate the IGLOo intervention (e.g. the toolkits) within the organisation and engage stakeholders with the approach. Table 1 shows a description of the intervention components for phase 1.

**Table 1:** Description of each intervention component for phases 1, 2 and 3

Phase	Intervention level			
	Individual—Worker with mental health condition	Group—Colleagues of the worker on sick leave	Leader—Line manager of the worker on sick leave	Organisation—HR, OH, Leaders responsible for sickness absence
Phase 1	N/A	N/A	N/A	3 × video presentations with supporting self-guided exercises and checklists: • Foundations: Mental health and RTW. • RTW policies and practices. • Engaging stakeholders to support a successful return to work. • Setting-up for success
Phase 2	Video presentations with supporting self-guided exercises and checklists supported by three coaching sessions: **Step 1:** Initial sick leave, what to do? **Step 2:** During sick leave, e.g. getting support, staying in contact with employer **Step 3:** Preparing for return to work **Step 4:** First weeks back at work, e.g. connecting with manager and colleagues **Step 5:** Keeping healthy and productive at work.	Workers on long-term sick leave and managers can share an information leaflet with colleagues which gives advice on how to support their colleague up to and during their absence.	Video presentations with supporting self-guided exercises and checklists: **Step 1:** initial sick leave. Prep-work, contacting the employee and follow up actions. **Step 2**: During sick leave, e.g. keeping in touch, using a co-ordinated approach. **Step 3:** Preparing for RTW. **Step 4:** First weeks back at work. Preparing for employee’s return and supporting them upon their return. **Step 5**: Staying healthy and productive at work. Regular check-ins with employee, etc.	N/A
Phase 3	**Step 6:** Job crafting to stay well in work. What is job crafting and how can it be used to the employee’s benefit?	Workers on long-term sick leave and managers can share an information leaflet with colleagues which gives advice on how to support their colleague reintegrate to the workplace upon their return.	**Step 6:** Job crafting to stay well in work. What is job crafting and how to support employee to make and manage work adjustments.	N/A

**Fig. 3:**
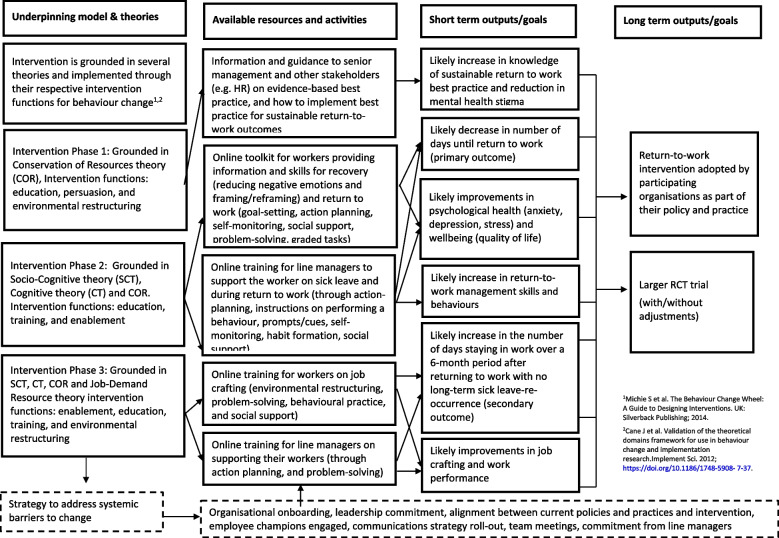
Logic model for the IGLOo pilot intervention study

*Phases 2 and 3* are directed towards the worker on long-term sick leave and their manager, targeting the individual, group and leader, and overarching levels of the IGLOo framework. There are two online toolkits hosted on a website: one aimed at the manager and the other at the worker. Both toolkits are self-led interventions used by the manager and the worker themselves. Grounded in the psychosocial theories mentioned above [22–25], the toolkits are designed to improve the worker/line manager relationship and increase self-efficacy and cognitive-affective wellbeing at six different steps of the workers’ RTW process: step (1) managing initial sick leave, step (2) during sick leave, step (3) preparing to RTW and step (4) first week back at work, step (5) staying healthy and productive at work and step (6) job crafting to stay well at work. Within each step, there are cognitive-behavioural-based tasks including self-led activities on problem-solving, goal setting and other practical tools (e.g. conversations checklists) with signposting to charities health services that provide mental health information and support. Additionally, the worker toolkit is supported by a workplace health coaching component consisting of up to three, 1-h-long telephone sessions with the research team. The coaching sessions are designed to support engagement with the intervention toolkit by encouraging the worker to use the worksheets in the toolkit to self-reflect on their thoughts, set goals and undertake actions to achieve their goals. The coaching will be delivered by three members of the research team of which two (AS and FM) are trained in workplace health coaching (www.centreforcoaching.com) and one holds a Post-Graduate Certificate in Low Intensity Psychological Interventions (OD). Table 1 provides a description of each intervention component for phases 2 and 3, whilst Fig. 3 outlines the logic model.

### Active control group

Organisations allocated into the control group will be asked to continue with their usual procedures and no training or guidance will be offered. Phase 2 and 3 participants in the control group will be asked to complete the same measurements as those in the phase 2 and 3 intervention group and at the same timepoints (phase 1 data will not be collected from the control group). At the end of the study, organisations in the control group will receive hard copies of the online toolkits.

### Preparing the workforce for the study

Prior to the start of any participant recruitment activities within each organisation, we will work closely with human resources staff, trade union representatives and worker representatives (e.g. workplace champions) to create a strong study communication strategy through for example, emails, posters, newsletters and team meetings. Communications will be disseminated at the start and throughout the study by maintaining refreshed timely messages to raise awareness and obtain buy-in from workers. We will also maintain regular monthly with human resources to building a strong working relationship with trust. Dates and times of these meetings will be recorded in the meeting minutes. These will be supplemented with researcher notes, including thoughts and observations about the organisation’s procedures and implementation approach. Collectively, these will provide information on the acceptability of the trial procedures including randomisation and trial measurements.

### Outcome measures

Outcome measures have been grouped as trial feasibility process outcome and research outcome measures (see below). Collected data and personal details will be stored according to the General Data Protection Regulation (GDPR, 2016/679) and research governance guidelines. A summary of the measures at the various time points is provided in Table 2. Survey data will be collected using Qualtrics and qualitative interview data will be collected either over the phone or using online conferencing facilities such as Microsoft Teams.

**Table 2:** Standard Protocol Items: Recommendations for Interventional Trials (SPIRIT) diagram illustrating the design and timescales of the IGLOo cluster-RCT pilot

Time point	Enrolment	Allocation	Baseline	3-month follow-up	6-month follow up	9-month follow-up	End of intervention (12-month)	End of study
**Enrolment**								
Organisation recruitment	x							
Eligibility Screen	x							
Organisation informed consent	x							
Allocation		x						
Manager informed consent			x					
Worker informed consent			x					
**Intervention**								
Leader e-learning (P1)			x					
Worker and manager toolkits (P2&3)			x	x	x	x	x	
Worker telephone coaching			x	x	x			
Control group—usual practice			x	x	x	x	x	
**Assessments**								
Organisational sickness policy	x							
Organisational sickness report data	x							
Feasibility outcomes (recruitment)			x					
Feasibility outcomes (retention, compliance)							x	x
Process evaluation	x		x	x	x	x	x	x
Intervention resource used								x
Economic evaluation	x		x	x	x	x	x	x
Organisation-wide survey		x						x
**Phase 1 leader measures**								
Timepoint			**Baseline**	**1 month**				
Sick leave and RTW policy and practice			x					
Intervention readiness			x	x				
Autonomy			x					
Demands			x					
Communication about mental health resources			x	x				
Demographics			x					
Intervention acceptability				x				
Intentions to apply learning				x				
Transfer of learning				x				
Intention to take part in phase 2				x				
Integration of intervention				x				
**Measures for worker on long-term sick leave**								
Demographics			x					
Days off sick			x	x**	x**	x**	x**	
Mental health			x	x	x	x	x	
Quality of life			x	x	x	x	x	
Healthcare resource used			x	x	x	x	x	
RTW self-efficacy			x	x	x	x	x	
Workplace support and communication			x	x	x	x	x	
Work adjustments			x	x	x	x	x	
Readiness to stay at work				x*	x*	x*	x*	
Job autonomy				x*	x*	x*	x*	
Job crafting				x*	x*	x*	x*	
Job satisfaction and intention to quit				x*	x*	x*	x*	
**Manager measures**								
Demographics			x					
Mental health management experience			x					
RTW management experience			x				x	
Work adjustments				x*	x*	x*	x*	
Work demands			x				x	
Autonomy			x				x	
Confidence managing sick leave and RTW			x	x	x	x	x	
RTW competency				x*	x*	x*	x*	
RTW competency (post- RTW)				x**	x**	x**	x**	

Key: x** = measures administered until employee returns to work; x* = measures administered once employee has returned to work; 1 month = measures administered at 1-month follow-up

### Trial feasibility and process-related outcome measures

The primary outcome of this pilot trial is to assess the feasibility of the research procedures (including recruitment, data collection, randomisation, acceptability of the intervention, retention and the presence of any adverse events) to inform the planning of a full RCT and test a full process evaluation methodology in advance of a fully powered trial. Specifically:

- The willingness of organisations, their workers on long-term sick leave and the line managers of those on long-term sick leave to take part in a 30-month study, and retention through follow-up (12 months) with intervention uptake and completion as primary endpoints.
- The potential for selection bias in control and intervention organisations as measured using participant characteristics at baseline.
- Intervention delivery, dose and fidelity whilst the worker is on long-term sick leave, and implementation of intervention delivery and adherence after the worker has returned to work.
- Acceptability of the intervention from different demographic groups (to understand what works for whom in which circumstances)
- Willingness and readiness of employers and their workers to adopt the proposed intervention in a manualised format (written as an instruction manual) that is flexible enough to meet individual and organisational needs in different settings.

The corresponding criteria for determining the success of the feasibility are outlined in the study outcome and data analysis section. The Integrative Process Evaluation Framework (IPEF) [34] will be used to evaluate the IGLOo study. The IPEF integrates the Reach, Effectiveness, Adoption, Implementation and Maintenance (RE-AIM) framework [35], and the Realist Evaluation theory [36] and has been developed to understand what works for whom in multilevel interventions such as those used in the proposed study. The RE-AIM framework evaluates five implementation outcomes: reach, effectiveness, adoption (e.g. delivery), implementation (intervention fidelity, costs and use) and maintenance (e.g. acceptability). The Realist Evaluation theory guides data generation to answer the question: “What works, for whom, in what respects, to what extent, in what contexts, and how?” Thus, allowing for the differential outcomes of the intervention for specific demographic groups such as gender and Black, Asian and Minority Ethnicity (BAME) to be identified. The IPEF is therefore a comprehensive five-phased framework guiding the collection, evaluation and reporting of process outcome data during the phases of a ‘real-world’ multi-level intervention: pre-intervention, intra-intervention, implementation and follow-up. The data collection methods outlined below capture the process outcome information using IPEF. In Realist evaluation, programme logic is formulated through CMO configurations. Figure 1 provides an overview of these hypothetical CMO configurations at each level of the IGLOo model. These CMO configurations will be empirically tested and refined based on our analyses.

#### Pre-study/pre-intervention start

Study reach will be assessed by collecting data on the number of organisations approached to participate in the study, number of expressions of interest and the number that consent to participate. Methods used to recruit these organisations will be documented and included in the evaluation to understand the underlying motivation of the organisation’s key stakeholders for participating in the study [37]. From the consenting organisations, we will collect long-term sickness absence data for the past 12 months (only total numbers and % by reasons), size of organisation and sector. We will also collect information from lead stakeholders (e.g. director of human resources) on their ‘readiness’ and expectations for the intervention. These data will help us identify potential *Context*ual barriers and facilitators to implementing the intervention.

Omnibus contextual factors (i.e. factors within the study which may inhibit or facilitate the extent to which the study is implemented) will be examined at the start of the study by collating views of the workforce through an organisational-wide online survey on workers’ knowledge of policies and practices around long-term sickness absence management and return to work support, perceptions of leaders commitment to supporting mental health and their communications for mental health support within the workplace [38]. This information will be used to examine leaders’ commitment to, and transparency of their commitment towards mental health conditions [39]. The survey will be sent out to control and intervention organisations at the start of the study. Discrete contextual factors (i.e. specific situational conditions that impact implementation) will be identified if and when they emerge throughout the pilot [40]. At the time of writing, there is upcoming industrial action in the UK in some of the sectors and we expect this, as well as other confounding social and economic factors, to influence participants’ experiences of the IGLOo intervention. We will therefore include questions about arising circumstances which may inhibit or facilitate implementation in the qualitative interviews with participants.

*Recruitment for phases 1 and 2*: information on the number of eligible staff for phase 1 (e.g. senior management team, HR staff) and the number consenting to take part will be collected in the intervention organisations only. For *phase 2* (workers on long-term sick leave and their managers), the number eligible to take part and the number consenting will be collected in both the control and intervention organisations. Reach will be assessed by comparing the overall eligible participant population invited to take part (the number of workers on long-term sick leave eligible for phase 2), with those who consent to participate (recruitment and reach are not assessed for phase 3 as all phase 2 participants automatically enter phase 3). Representativeness of the participant population will be assessed from information obtained through organisational records and will be compared against demographic information from the baseline questionnaires of consenting workers.

#### Intra-intervention and implementation

Guided by the IPEF, we will assess the implementation of intervention activities evaluation for each intervention phase. Intervention dose and compliance will be assessed through analytics data, including number accessing online e-learning or toolkits, number of downloads of resources, completion of modules or steps and downloads of self-guide exercises. Acceptability and usability of the intervention will be assessed using questionnaires and interviews at the end of each intervention phase. The number of coaching sessions attended by phase 2/3 worker participants will be documented, and a random selection of coaching session will be audio-recorded to assess fidelity. We will ask participants to send us screenshot/copies of completed learning activities, checklists, goal-setting and action-planning sheets and other record sheets.

Structured interviews will be conducted with all participants at the end of each intervention phase with a view to explore the mechanisms by which the intervention works. For example, we will explore how participants have attempted to transfer knowledge, skills and methods learned from each intervention phase within their respective roles (e.g. as a leader implementing policies, as a line manager supporting a worker on sick leave, and as a worker requesting work adjustments). Questionnaires and interviews will also be used to understand intentions and motivations for intervention engagement, changes to cognitions and behaviours as a result of the intervention, and any contextual factors (barriers and facilitators) that may have influenced engagement with the intervention materials.

#### End of study

At the end of the 30-month study participation, we will interview control and intervention employers (e.g. human resources/senior managers) to explore their perceptions of the study including data collection methods and time commitments. To evaluate maintenance, we will also explore interest in on-going usage of the intervention materials and whether the intervention could form part of their return-to-work policies and processes moving forward. Finally, we will re-examine the omnibus contextual factors in control and intervention organisations by repeating organisational-wide online survey.

### Main trial research-related outcomes

We will collect the following data to inform the feasibility of the pilot study as a whole.

#### Phase 1 measures

For phase 1 participants, the outcome data will be collected using questionnaires at baseline (pre-intervention) and post-intervention approximately 1–2 months after being given access to the e-learning resources. Information collected at baseline will include questions about awareness of current policies and procedures, job autonomy, job demands (Health and Safety Executive Job Demands Scale) [41], demographic information including occupational classification (Standard Occupational Classification, 2020) [42] intervention readiness and communication about mental health and resources. The latter two questionnaires will be asked again at 1-month follow-up.

#### Primary outcome measures (phases 2 and 3)

The primary research outcome of the study, the total number of days on sick leave, will be collected for control and intervention participants. The number of sick leave days will be calculated from the first day of sick leave recorded on a participant’s ‘fit note’ (obtained from their general practitioner or medical doctor and submitted to their employer), until their first day back at work using self-report data from the participant. Sick leave days are counted in calendar days and not just the days a person typically works on. For example, weekends and non-working days are counted as part of sick days. The information will be collected from participants via text messaging at monthly intervals as well as asked in the baseline questionnaire, and in their 3-, 6-, 9- and 12-month follow-up questionnaires. Participants will be asked whether they have returned to work and, if so, what was the date of their first day back at work. If they have not returned to work during their participation, then the last point this question will be asked will be at their final data collection point at 12 months (see Table 2). Self-report of sickness absence has been shown to be adequately reliable when compared with organisational records [43, 44].

#### Secondary outcome measures for worker participants on long-term sick leave

##### The number of days staying at work over a 6-month period with no exit or long-term sick leave re-occurrence (main trial secondary outcome)

Once a participant is back at work, we will collect regular data from them (through monthly texts) on any subsequent sick leave taken or leaving the organisation over a 6-month period.

The following secondary outcome measures will be collected via questionnaires at baseline, 3, 6, 9 and 12 months from participant workers.

##### Mental health and quality of life measures

The Patient Health Questionnaire (PHQ-9) [45], a nine-item questionnaire, will be used to measure depression. Anxiety will be measured by the 7-item General Anxiety Disorder Questionnaire (GAD-7) [46], and burnout will be measured using the 3-item exhaustion scale from Utrecht Burnout Scale [47]. The EURO-QOL [48] questionnaire will be used to assess quality of life. Self-report information on the NHS and social care mental health and wellbeing services and employer mental health and wellbeing services will be collected [49].

##### Return to work confidence and competence

Expectations about the length of sick leave will be asked using one question, “for how long do you believe you will be on sick leave from today?”. The 11-item Return-to-work Self-Efficacy Scale [50] will be used to assess worker’s confidence to return to work. Sixteen items from the line manager competency questionnaire [51] will be used to assess worker’s perceptions of their line manager’s competence in managing the worker’s sickness absence and return to work. An identical set of questions from this questionnaire will be used with the manager to identify their actions and behaviours from their own perspective.

##### Psychosocial work measures

Three items from the Manager Communication Questions [52] will assess participant’s confidence in communicating health matters with their managers, and two items will assess communication satisfaction. Colleague support will be measured by 1 item [53], and 5 items will assess manager support [54]. Two items will assess the worker’s intention to quit [55], and the 4-item job autonomy question will be used from the Basic Psychological Needs Satisfaction at Work Scale [56, 57]. The 9-item Readiness to Stay at Work Scale [58] will be used to assess a participant’s readiness to stay in their role at work and the 15-item Job Crafting Questionnaire [59, 60] will assess the changes workers make to their job tasks. Work Productivity will be assessed by a single question asking workers about their relative productivity in the past week. A one-item job satisfaction scale will be used to assess satisfaction [61]. A four-item scale created by the research team will be used to assess the work adjustments made for the workers when they return to work.

##### Demographic information

Workers will be asked to provide demographic information including age, gender, ethnicity, job role and tenure at baseline.

#### Secondary outcome measures for managers

The following secondary outcome measures will be collected via questionnaires at baseline, 3, 6, 9 and 12 months from participant managers.

##### Experience confidence and competence with managing mental health and RTW

At baseline only, managers will be asked about their experience as a manager (three questions), any RTW training received related to long-term sickness absence (three questions) and a six-item questionnaire on the return-to-work climate within the organisation (six questions). Managers will also be asked about their confidence in managing mental health issues and promoting a mentally healthy workplace using six items [62]. Sixteen items from the manager competency scale [51] will be used to identify the actions and behaviours undertaken by the manager when supporting the worker during their sick leave and return to work. A four-item scale created by the research team will be used to assess the work adjustments made for the workers when they return to work.

##### Autonomy and job demands

Line managers will also be asked about work autonomy that will be assessed on a four-item scale [56, 57] and job demands [54].

##### Demographic information

Managers will be asked to provide demographic information on age, gender, ethnicity, job role and tenure at baseline.

### Participant appreciation

As a token of our gratitude for taking the time to complete the organisation-wide surveys, each participant will be asked if they would like to opt-in to a randomly selected, organisation-wide prize draw to win a voucher worth £50.00. For phases 2 and 3, all participants who complete all five of the worker questionnaires or all five of the manager questionnaires will be offered the opportunity to enter a further draw to win another £50 Amazon voucher. There will be two draws for the organisation-wide survey, a draw for managers and a draw for workers (four in total per organisation).

### Study outcome and data analysis

The primary outcome of this pilot trial is to determine its feasibility for a main trial. This decision will be made using a traffic light system using predefined stop-go criteria: green light—main trial feasible; amber light—main trial feasible with adjustments; and red light—main trial should not proceed. The four criteria contributing to the traffic light system include (1) recruitment rate (green light: ≥ 50% of participants invited; amber: 25–49%; red: < 25%); (2) retention (green: ≥ 70%; amber 40–69%; red: < 40%); (3) intervention completion (green: ≥ 50%; amber: 25–49%; red < 25% ); and (4) usability of intervention materials (green: completion of all intervention components ≥ 50; amber: 25–49%; red: < 25%). An RCT to study the effectiveness of the intervention will be considered feasible when all the green criteria are met. If not, adjustments for the study protocol will be formulated for amber criteria. If red criteria are met in all four points above, a full RCT will not proceed.

### Process evaluation analysis plan

Process evaluation analyses will be guided by IPEF. Quantitative process data will primarily take the form of simple descriptive statistics (e.g. proportions and percentages, means and standard deviations). Qualitative interview data will be transcribed verbatim and analysed using inductive thematic analysis procedures. Quantitative and qualitative data will be triangulated and systematically coded using the core theoretical frameworks to identify the change mechanisms of how leaders, workers and line managers translate the intervention materials into behaviour changes and the barriers and facilitators in doing so. This will provide us with valuable information on what works and under what context (e.g. readiness for change, culture) and how these lead to our intended outcomes.

#### Statistical analysis plan

A statistical analysis plan for the pilot will be finalised and agreed prior to analysis by the research team. The research outcome measures will be analysed and reported according to the Consolidation Standards of Reporting (CONSORT) statement for cluster RCTs. As this is a pilot study, we will examine the primary and secondary research outcomes to mimic practice for a full trial in addition to finalising the sample size for a definitive main trial. However, as this is a pilot study, no emphasis will be placed on the statistical significance of the effects.

The primary analyses will compare the number of calendar sick leave days (including non-working days) until the first day of partial or full return to work between arms post-12-month baseline data collection. Results will be treated as preliminary and interpreted with caution. Statistical analysis will be carried out on an intention-to-treat basis with missing outcome data being imputed using multiple imputation by chained equations (MICE) [63]. Effect sizes will be calculated but no emphasis will be put on the *p* values for any inferential statistical tests conducted. In order to inform the sample size calculation for a future trial, the present study analysis will carry out a preliminary examination of (1) between-group effect sizes and (2) the magnitude of the intra-cluster correlation coefficient (ICC) of the primary outcome using a mixed effects model.

In a future main trial, we will conduct a mixed effects Cox regression, in which censoring allows all data available at all time points to be used and account for missing data and clustering effect, to estimate a two-sided 95% confidence interval (CI) to show a reliable range for the true difference in the primary outcome (i.e. number of days taken to return to work [partial or full] between the intervention and control arms). Analysis of the main secondary outcome (number of days staying in work since RTW, with no exit or long-term sick leave re-occurrence) and other secondary outcomes (e.g. mental health, RTW self-efficacy, job crafting) 3, 6, 9 and 12 months will be analysed using a similar modelling strategy, but with the type of model adjusted for the nature of the outcome variable, and assumptions checked as far as possible to verify that the model types (e.g. mixed effects linear models, mixed effects, Cox regression models) appear correct. Additional analyses will focus on comparing the baseline characteristics of intervention and control arms as well as those lost to follow-up with those not lost to follow-up to assess for bias. As a future full trial analysis will control for appropriate demographic and work-related factors, exploratory analysis of pilot data will be used to identify which covariates should be included.

#### Economic evaluation plan

Economic analysis will be exploratory using data collected by the EuroQoL-5DL (EQ-5DL) quality of life measure for the quality-adjusted life years (QALYs) and the use of health services or medical treatment collected by the Health and Wellbeing Services questionnaire [49]. Intervention delivery costs (delivering the intervention components, website build and delivery, training delivery including line manager’s time) will also be collected.

### Pilot trial management, monitoring and governance

All data will be anonymised and entered into a secure database and only accessible by the research team and authorised personnel. The study will comply with the Data Protection Act which requires data to be anonymised as soon as it is practical to do so. Personal data will be processed on a public task basis under the General Protection Regulation (GDPR). Participant personal data will be stored confidentially, and their participation will not be shared with their organisation. The pilot trial will be coordinated by the trial management group (principal investigator, co-investigators, study researcher, Patient and Public Involvement (PPI) group lead) in conjunction with the NIHR and members of policy stakeholders, employer representatives and PPI group. A Trial Steering Committee (TSC) consists of the principal investigator, an independent chair, two external members, two employer representatives and a statistician. As this is a pilot study, no Data Monitoring Committee will be formed. The integrity of data entry will be ensured using a trial-specific standard operating procedure (Trial Master File, Rotherham, Doncaster and South Humber NHS Trust).

### Dissemination plan

It is anticipated that the findings of this study will be published in peer-reviewed journals and professional journals, presented at conferences and other outlets, and that the results will be disseminated to all participating organisations and study participants who wish to be informed.

## Discussion and conclusion

This article outlines the protocol for a study examining the feasibility and acceptability of conducting and evaluating a randomised controlled trial of a multi-component, multi-phase RTW intervention. The intervention is novel in its design, and in its importance for addressing the need of employers in managing long-term sick leave and RTW for workers from a mental health perspective.

Early intervention to support the RTW of workers holds economic and social significance. For the worker and their family, work provides income, structure and social connections [11]. For the employer, it helps to reduce costs associated with long-term sick leave and enable retention of knowledge and culture of wellbeing. The IGLOo framework [17] synthesised on best practice research and current psychosocial theories (CoR, JDR, SCT and CT) [22–25] proposes different resources that can be utilised by the Individuals, the Leaders, the Group and the Organisation as a whole. It also addresses the overarching societal context, organisational policies and government legislature, in order to promote sustainable RTW. Poor mental wellbeing accounts for a substantial proportion of long-term sick leave [1]. Evidence shows that those who are absent from work for at least 6 months are unlikely to return to employment [11]. One shortcoming of existing RTW research is the lack of focus on contextual factors prior to the RTW of workers with common mental health problems. Understanding these contextual factors could help us to understand how to prevent sick-leave relapse following a worker’s RTW [34, 64]. Our comprehensive process evaluation framework aims to address this gap.

Overall, the IGLOo trial takes the conceptualisation of the IGLOo framework [17] and translates into a working intervention, with the aim to test the feasibility of the intervention in the real-world environment, with a view to inform a large-scale trial in the future.

## Data Availability

The datasets generated during the study will be available from the corresponding author on reasonable request.

## Abbreviations

UK: United Kingdom
IGLOo: Individual, Group, Leader, Organisation and Over-arching context
RCT: Randomised controlled trial
RTW: Return to work
COVID-19: Coronavirus SARS-2
NICE: National Institute for Health and Care Excellence
CBT: Cognitive behavioural therapy
SMD: Standardised mean difference
CI: Confidence interval
HR: Human resources
CT: Cognitive theory
CAT: Communication Accommodation Theory
SCT: Socio-Cognitive Theory
CoR: Conservation of Resources
JDR: Job Demands—Resources
ISRCTN: International Standard Randomised Controlled Trial Number
NHS: National Health Service
PPI: Patient and Public Involvement
SME: Small and Medium Enterprises
LE: Large Enterprises
GDPR: General Data Protection Regulation
IPEF: Integrated Process Evaluation Framework
RE-AIM: Reach, Effectiveness, Adoption, Implementation and Maintenance
CMO: Context Mechanism Outcome
BAME: Black, Asian and Minority Ethnic
HSE: Health and Safety Executive
SOC: Standard Occupational Classification
PHQ-9: Patient Health Questionnaire—9 items
GAD-7: Generalised Anxiety Disorder—7 items
UBS: Utrecht Burnout Scale
EURO-QOL: EuroGroup Quality of Life
QALYs: Quality-adjusted life years
CONSORT: Consolidation Standards of Reporting
MICE: Multiple Imputation by Chained Equations
ICC: Intra-cluster Correlation Coefficient
NIHR: National Institute for Health Research
TSC: Trial Steering Committee

## References

[1] Deloitte. Mental health and employers. Refreshing the case for investment.2020. https://www2.deloitte.com Accessed 15th November 2020.

[2] National Institute of Care and Excellence (NICE). Workplace health: long-term sickness absence and capability to work. Report [NG146]. 2019. https://www.nice.org.uk/guidance/ng146 Accessed 8th Dec 2021.

[3] Department of Work and Pensions/Department of Health and Social Care. The employment of disabled people 2019. https://assets.publishing.service.gov.uk/government Accessed 5^th^ June 2020

[4] Stevenson D, Farmer P. Thriving at work: Independent review of mental health and employers. 2017. https://assets.publishing.service.gov.uk/government Accessed 8^th^ Dec 2021

[5] British Medical Association. Ageing and the workplace. 2016. https://www.bma.org.uk/media/427 Accessed 15th November 2020.

[6] Kathirvel N (2020). Post COVID-19 pandemic mental health challenges. Asian J Psychiatr..

[7] Nochaiwong S, Ruengorn C, Thavorn K, Hutton B, Awiphan R, Phosuya C, Ruanta Y, Wongpakaran N, Wongpakaran T (2021). Global prevalence of mental health issues among the general population during the coronavirus disease-2019 pandemic: a systematic review and meta-analysis. Sci Rep..

[8] Penninx BW, Benros ME, Klein RS, Vinkers CH (2022). How COVID-19 shaped mental health: from infection to pandemic effects. Nat Med..

[9] Black C, Frost D. Health at work – an independent review of sickness absence. 2011. https://assets.publishing.service.gov.uk/government Accessed 15th November 2020.

[10] Cameron J, Sadlo G, Hart A, Walker C (2016). Return-to-work support for employees with mental health problems: Identifying and responding to key challenges of sick leave. Br J Occup Ther..

[11] Waddell G, Burton KA. Is work good for your health and well-being? 2006. http://www.mas.org.uk/uploads/artlib/is-work-good-for-your-health-and-well-being.pdf Accessed 15th November 2020.

[12] Knudsen AK, Harvey SB, Mykletun A, Øverland S (2013). Common mental disorders and long-term sickness absence in a general working population. The Hordaland Health Study. Acta Psychiat Scand..

[13] Melkevik O, Clausen T, Pedersen J, Garde AH, Holtermann A, Rugulies R (2018). Comorbid symptoms of depression and musculoskeletal pain and risk of long term sickness absence. BMC Public Health..

[14] Naylor C, Parsonage M, McDaid D, KNapp M, Fossey M, Galea A. Long-term conditions and mental health. 2012. https://www.kingsfund.org.uk Accessed 9^th^ November 2020.

[15] Mikkelsen MB, Rosholm M (2018). Systematic review and meta-analysis of interventions aimed at enhancing return to work for sick-listed workers with common mental disorders, stress-related disorders, somatoform disorders and personality disorders. Occup Environ Med..

[16] Etuknwa A, Daniels K, Eib C (2019). Sustainable return to work: a systematic review focusing on personal and social factors. J Occupl Rehabil..

[17] Nielsen K, Yarker J, Munir F, Bültmann U (2018). IGLOO: an integrated framework for sustainable return to work in workers with common mental disorders. Work Stress..

[18] Munir F, Yarker J, Hicks B, Donaldson-Feilder E (2012). Returning employees back to work: developing a measure for supervisors to support return to work (SSRW). J Occupl Rehabil..

[19] Nieuwenhuijsen K, Verbeek JH, Neumeyer-Gromen A, Verhoeven AC, Bültmann U, Faber B. Interventions to improve return to work in depressed people. Cochrane Database Syst Rev. 2020;(10) 10.1002/14651858.CD006237.pub4. 10.1002/14651858.CD006237.pub4 PMC8094165 33052607

[20] Nigatu YT, Liu Y, Uppal M, McKinney S, Rao S, Gillis K, Wang J (2016). Interventions for enhancing return to work in individuals with a common mental illness: systematic review and meta-analysis of randomized controlled trials. Psychol Med..

[21] Philpot DR, Gavrilova AM (2021). Post-Leave (Return to Work) Training Needs and Human Resource Development. Adv Dev Hum Resour..

[22] Hobfoll SE (1989). Conservation of resources: A new attempt at conceptualizing stress. Am Psychol..

[23] Beck AT (1993). Cognitive theory and therapy: past, present and future. J Consult Clin Psychol..

[24] Giles H, Coupland N, Coupland IU (1991). *Accommodation theory: communication, context, and consequence*.

[25] Bandura A, Freeman WH, Lightsey R (1999). Self-efficacy: the exercise of control.

[26] Craig P, Dieppe P, Macintyre S, Michie S, Nazareth I, Petticrew M (2008). Developing and evaluating complex interventions: the new Medical Research Council guidance. BMJ..

[27] City population data. The population of the boroughs in the Metropolitan County of South Yorkshire. https://www.citypopulation.de/en/uk/southyorkshire. Accessed 16 Jan 2022.

[28] Office for National Statistics. Census: Population Estimates for the United Kingdom. 2021. https://www.ons.gov.uk/peoplepopulationandcommunity/populationandmigration/populationestimates Accessed 22^nd^ July 2021.

[29] Nomis. Official labour market statistics. 2020. https://www.nomisweb.co.uk/ Accessed 9^th^ November 2020.

[30] Office for Health Improvement and Disparities: Health Profile for Yorkshire and Humber 2021. 2021. https://fingertips.phe.org.uk/static-reports/health-profile-for-england/regional-profile-yorkshire_and_the_humber.html Accessed 22^nd^ July 2021.

[31] Bambra C, Munford L, Alexandros A, Barr B, Brown H, Davies H, Konstantinos D, Mason K, Pickett K, Taylor C, Taylor-Robinson D. COVID-19 and the Northern Powerhouse: tackling inequalities for health and productivity. 2020. https://www.thenhsa.co.uk Accessed 19^th^ February 2021.

[32] Eldridge SM, Costelloe CE, Kahan BC, Lancaster GA, Kerry SM (2016). How big should the pilot study for my cluster randomised trial be?. Stat Meth Medical Res..

[33] Teare MD, Dimairo M, Shephard N, Hayman A, Whitehead A, Walters SJ (2014). Sample size requirements to estimate key design parameters from external pilot randomised controlled trials: a simulation study. Trials..

[34] Nielsen K, De Angelis M, Innstrand ST, Mazzetti G (2023). Quantitative process measures in interventions to improve employees’ mental health: a systematic literature review and the IPEF framework. Work Stress..

[35] Glasgow RE, Vogt TM, Boles SM (1999). Evaluating the public health impact of health promotion interventions: the RE-AIM framework. Am J Pub Health..

[36] Pawson R, Tilley N (1997). Realistic Evaluation| SAGE Publications Inc.

[37] Nielsen K, Randall R (2013). Opening the black box: presenting a model for evaluating organizational-level interventions. Eur J Work and Organ Psychol..

[38] Dimoff J, Kelloway EK. Mental health problems are management problems: exploring the critical role of managers in supporting employee mental health Organ Dynamics. 2019; 48(3):105-112.10.1016/j.orgdyn.2018.11.003

[39] Dimoff JK, Kelloway EK (2019). With a little help from my boss: the impact of workplace mental health training on leader behaviors and employee resource utilization. J Occup Health Psych..

[40] Nytrø K, Saksvik PØ, Mikkelsen A, Bohle P, Quinlan M (2000). An appraisal of key factors in the implementation of occupational stress interventions. Work Stress..

[41] Health and Safety Executive. The Management Standards. https://www.hse.gov.uk/stress/standards/ Accessed 7^th^ May 2019

[42] Office for National Statistics. Standard Occupational Classification. 2020. https://www.ons.gov.uk/methodology/classificationsandstandards/standardoccupationalclassificationsoc/soc2020/soc2020volume1structureanddescriptionsofunitgroups

[43] Ferrie JE, Kivimäki M, Head J, Shipley MJ, Vahtera J, Marmot MG (2005). A comparison of self-reported sickness absence with absences recorded in employers’ registers: evidence from the Whitehall II study. Occup Environ Med..

[44] Johns G, Miraglia M (2015). The reliability, validity, and accuracy of self-reported absenteeism from work: a meta-analysis. J Occup Health Psychol..

[45] Kroenke K, Spitzer RL, Williams JB (2001). The PHQ-9: validity of a brief depression severity measure. J Gen Intern Med..

[46] Spitzer RL, Kroenke K, Williams JB, Löwe B (2006). A brief measure for assessing generalized anxiety disorder: the GAD-7. Arch Intern Med..

[47] Schaufeli WB, Van Dierendonck D (2000). Utrechtse Burnout Schaal (UBOS), Handleiding [Utrecht Burnout Scale, Manual].

[48] The EuroQol Group (1990). EuroQol-a new facility for the measurement of health-related quality of life. Health Policy..

[49] Peveler R, Kendrick A, Buxton M, Longworth L, Baldwin D, Moore M, Chatwin J, Goddard J, Thornett A, Smith H, Campbell M (2005). A randomised controlled trial to compare the cost-effectiveness of tricyclic antidepressants, selective serotonin reuptake inhibitors and lofepramine. Health Tech Assess..

[50] Lagerveld SE, Blonk RWB, Brenninkmeijer V, Schaufeli WB (2010). Return to work among employees with mental health problems: development and validation of a self-efficacy questionnaire. Work Stress..

[51] Yarker J, Munir F, Donaldson-Feilder E, Hicks B (2010). Managing rehabilitation: a competency framework for managers to support return to work.

[52] Yarker J. 6-item workplace communication questionnaire. Available from https://www.affinityhealthatwork.com upon request. 2019. Accessed 6 Mar 2023.

[53] Vornholt K, Uitdewilligen S, van Ruitenbeek G, Zijlstra F (2021). The development and validation of the workplace acceptance scale: evidence from a sample of workers with disabilities. J Vocat Rehabil..

[54] Health and Safety Executive. The Management Standards. No date. Accessed 20^th^ September 2022. https://www.hse.gov.uk/stress/standards/downloads.htm

[55] Bentein K, Vandenberghe C, Vandenberg R, Stinglhamber F (2005). The role of change in the relationship between commitment and turnover: a latent growth modeling approach. J Applied Psychol..

[56] Deci EL, Ryan RM (2000). The" what" and" why" of goal pursuits: human needs and the self-determination of behavior. Psychol Inq..

[57] Deci EL, Ryan RM, Gagné M, Leone DR, Usunov J, Kornazheva BP (2001). Need satisfaction, motivation, and well-being in the work organizations of a former eastern bloc country: a cross-cultural study of self-determination. Pers Soc Psychol Bull..

[58] Franche RL, Corbière M, Lee H, Breslin FC, Hepburn CG (2007). The readiness for return-to-work (RRTW) scale: development and validation of a self-report staging scale in lost-time claimants with musculoskeletal disorders. J Occup Rehabil..

[59] Nielsen K, Abildgaard JS (2012). The development and validation of a job crafting measure for use with blue-collar workers. Work Stress..

[60] Nielsen K, Antino M, Sanz-Vergel A, Rodríguez-Muñoz A (2017). Validating the Job Crafting Questionnaire (JCRQ): a multi-method and multi-sample study. Work Stress..

[61] Nagy MS (2002). Using a single-item approach to measure facet job satisfaction. J Occup Organ Psychol..

[62] Gayed A, LaMontagne AD, Milner A, Deady M, Calvo RA, Christensen H, Mykletun A, Glozier N, Harvey SB (2018). A new online mental health training program for workplace managers: pre-post pilot study assessing feasibility, usability, and possible effectiveness. JMIR Ment Health..

[63] Azur MJ, Stuart EA, Frangakis C, Leaf PJ (2011). Multiple imputation by chained equations: what is it and how does it work?. Int J Methods Psychiatr Res..

[64] Nielsen K, Randall R (2012). The importance of employee participation and perceptions of changes in procedures in a teamworking intervention. Work Stress..

